# Structure-Based Virtual Screening and In Silico Evaluation of Marine Algae Metabolites as Potential α-Glucosidase Inhibitors for Antidiabetic Drug Discovery

**DOI:** 10.3390/ph19010098

**Published:** 2026-01-05

**Authors:** Bouchra Rossafi, Oussama Abchir, Fatimazahra Guerguer, Kasim Sakran Abass, Imane Yamari, M’hammed El Kouali, Abdelouahid Samadi, Samir Chtita

**Affiliations:** 1 Laboratory of Analytical and Molecular Chemistry, Faculty of Sciences Ben M’Sik, Hassan II University of Casablanca, Casablanca 20100, Morocco; bouchrarossafi7@gmail.com (B.R.); oussamaabchir12@gmail.com (O.A.); guerguerfatimazahra@gmail.com (F.G.); yamariimane86@gmail.com (I.Y.); m.elkouali.fsbm@gmail.com (M.E.K.); 2 Department of Physiology, Biochemistry and, Pharmacology, College of Veterinary Medicine, University of Kirkuk, Kirkuk 36001, Iraq; kasim.s.abass@uoalkitab.edu.iq; 3 Department of Chemistry, College of Science, United Arab Emirates University, Al Ain P.O. Box 15551, United Arab Emirates

**Keywords:** diabetes mellitus, alpha-glucosidase, seaweed metabolites, virtual screening approach

## Abstract

**Background/Objectives:** Diabetes mellitus is a serious global disease characterized by chronic hyperglycemia, resulting from defects in insulin secretion, insulin action, or both. It represents a major health concern affecting millions of people worldwide. This condition can lead to severe complications significantly affecting patients’ quality of life. Due to the limitations and side effects of current therapies, the search for safer and more effective antidiabetic agents, particularly from natural sources, has gained considerable attention. This study investigates the antidiabetic potential of seaweed-derived compounds through structure-based virtual screening targeting α-glucosidase. **Methods**: A library of compounds derived from the Seaweed Metabolite Database was subjected to a hierarchical molecular docking protocol against α-glucosidase. Extra Precision (XP) docking was employed to identify the top-ranked ligands based on their binding affinities. Drug-likeness was assessed according to Lipinski’s Rule of Five, followed by pharmacokinetic and toxicity predictions to evaluate ADMET properties. Density Functional Theory (DFT) calculations were performed to analyze the electronic properties and chemical reactivity of the selected compounds. Furthermore, molecular dynamics simulations were carried out to examine the stability and dynamic behavior of the ligand–enzyme complexes. **Results:** Following XP docking and ADMET prediction, four promising compounds were selected: Colensolide A, Rhodomelol, Callophycin A, and 7-(2,3-dibromo-4,5-dihydroxybenzyl)-3,7-dihydro-1*H*-purine-2,6-dione. Molecular dynamics simulations further confirmed the structural stability and strong binding interactions of these compounds within the α-glucosidase active site. **Conclusions:** This investigation demonstrated the important role of seaweed-derived compounds in inhibiting α-glucosidase activity. Further experimental validation is warranted to confirm their biological activity and therapeutic potential.

## 1. Introduction

Diabetes mellitus is a chronic metabolic disorder characterized by persistent hyperglycemia resulting from a deficiency in insulin secretion, insulin action, or both [1,2]. It is primarily classified into two main types: type 1 diabetes, which is of autoimmune origin [3], and type 2 diabetes, which is associated with insulin resistance often linked to environmental factors such as sedentary lifestyle, poor diet and obesity, with the second type is the most common form, accounting for 90% of all cases. Furthermore, the symptoms of diabetes can differ depending on the type and individual factors, but common manifestations include frequent urination, excessive thirst, unexplained weight loss, fatigue, blurred vision, and numbness or tingling in the extremities [4,5]. According to the International Diabetes Federation (IDF), approximately 532 million people worldwide were diagnosed with diabetes in 2021, a number projected to rise to 783 million by 2045 [6]. This steadily increasing prevalence makes diabetes a major global public health issue. Moreover, in the absence of adequate management, diabetes can lead to severe complications affecting multiple organs [7], including cardiovascular diseases, diabetic nephropathy, retinopathy, and neuropathy [8,9]. Given these serious consequences, there is a pressing need to develop novel antidiabetic therapies that can improve glycemic control and enhance patients’ quality of life.

In this context, diabetes management largely focuses on the regulation of postprandial blood glucose levels. To this end, digestive enzyme inhibitors, particularly alpha-glucosidase inhibitors, have been widely used to slow the breakdown of complex carbohydrates into simple sugars [10,11,12]. However, existing drugs such as Acarbose, Miglitol, and Voglibose are often associated with gastrointestinal side effects, including flatulence, abdominal discomfort, and diarrhea [13,14,15]. These limitations have prompted an active search for new alpha-glucosidase inhibitors, especially those derived from natural sources [16], with the aim of achieving better efficacy, improved safety profiles, and higher patient tolerance.

The drug discovery process is widely recognized as being lengthy, costly, and resource-intensive, often requiring several years of research and substantial financial investment before identifying an effective therapeutic candidate [17,18]. To overcome these limitations, computer-aided drug design (CADD) approaches have emerged as powerful tools to accelerate the early stages of drug development [19,20]. Among them, structure-based virtual screening (SBVS) allows for the rapid evaluation of large compound libraries against a specific biological target [21,22,23]. This strategy helps to reduce experimental costs and focuses research efforts on the most promising candidates, while enhancing both efficiency and precision [24].

In the recent years, natural products have attracted considerable attention in drug discovery due to their structural diversity, biological relevance, and favorable pharmacokinetic properties [25,26]. Among them, compounds derived from marine organisms have shown promising therapeutic potential in the treatment of various diseases [27,28]. Marine environments, in particular, are a rich and underexplored source of bioactive metabolites, offering unique chemical scaffolds [29]. Among marine sources, algae stand out as a significant group, known for their diverse biochemical composition including polysaccharides, polyphenols, carotenoids, and peptides [30,31]. These metabolites exhibit multiple pharmacological activities, such as antioxidant, anti-inflammatory, and anticancer effects [32,33], making them attractive candidates for the development of new antidiabetic agents. Compared to terrestrial plants or other natural sources, marine algal metabolites often contain unique chemical features, such as halogenated compounds and sulfated polysaccharides, which are rarely found in land-based organisms. These structural characteristics not only increase the chemical diversity of marine algae but may also enhance their interaction with biological targets, improving binding affinity. Given their pharmacological value and chemical diversity, evaluating such molecules through in silico approaches could provide insights and support their valorization in therapeutic contexts.

Despite the growing number of studies reporting natural bioactive compounds as potential α-glucosidase inhibitors, the novelty of the present work lies in the use of a database specifically composed of natural compounds derived from marine algae, which was used and explored for the first time against α-glucosidase for the treatment of diabetes. In the present study, a structure-based virtual screening was performed targeting the alpha-glucosidase enzyme to identify novel inhibitors with promising pharmacological profiles. For this purpose, a custom library of approximately 1191 seaweed metabolites was compiled. A comprehensive set of in silico techniques was employed to thoroughly evaluate the lead candidates, including molecular docking to predict binding affinity and interaction modes, pharmacokinetic and pharmacodynamic property prediction to assess drug-likeness and ADMET profiles, and molecular dynamics simulations to investigate the stability of the protein–ligand complexes [34]. Additionally, quantum chemical calculations were conducted to analyze the molecular reactivity and electronic properties of the lead compounds [35], providing further insight into their potential as effective alpha-glucosidase inhibitors.

## 2. Results and Discussion

### 2.1. Validation of Molecular Docking Protocol

**Figure 1:**
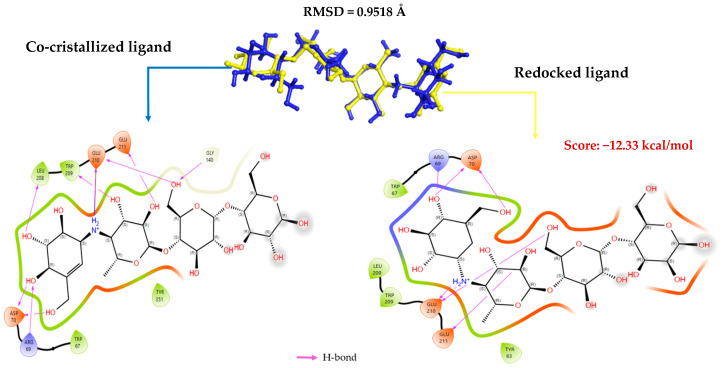
Superposition of the redocked ligand (in yellow) with the co-crystallized ligand (in blue) and comparison of the interactions formed with the residues of the active site.

The reference drug Acarbose was redocked into the target protein 2F6D’s active site in order to confirm the molecular docking procedure employed in this investigation. The purpose of this process is to evaluate the docking software’s capacity to replicate the crystallized ligand’s experimental conformation. For the redocking, Glide’s XP mode was used. After being removed from the crystallographic complex, the natural ligand was redocked into the defined binding site. The accuracy of the redocking was evaluated by calculating the Root Mean Square Deviation (RMSD) value between the redocked ligand and the co-crystalized ligand. An RMSD value of 0.9518 Å was obtained, which is below 2 Å, confirming the reliability of the docking protocol in accurately predicting the ligand’s position and orientation within the active site. Therefore, this step validates the use of the protocol for virtual screening of the other compounds. An analysis of the interactions formed between the redocked ligand and the residues of the active site was conducted (Figure 1). This analysis allowed for a comparison of the interactions established during redocking with those observed in the crystallographic structure of the native complex. It was observed that the redocked ligand retains the majority of the key interactions, and the consistency of these interactions with those observed in the original structure reinforces the validity of the docking protocol. This observation also allows us to conclude that the adopted protocol is capable of faithfully reproducing the interaction conditions between the ligand and the target.

### 2.2. Virtual Screening Analysis

To identify potential inhibitors targeting the active site of the protein, a hierarchical virtual screening protocol was employed using the Glide module of Schrödinger. This approach consisted of three sequential docking steps with increasing levels of precision: HTVS, SP, and XP.

All compounds were docked specifically into the defined active site of the protein. During the HTVS docking stage, the compounds were subjected to a rapid screening algorithm aimed at efficiently exploring their potential binding poses within the active site. The top 10 percent of the best docked ligands based on HTVS scores were selected for further analysis. In the next step, SP docking allowed for a more thorough sampling of molecular conformations. The preselected ligands were redocked with increased precision, in order to refine their binding poses and obtain scores more representative of their affinity with the target. Finally, the most promising compounds, top 10 percent of SP-docked ligands were subjected to the final stage of XP docking. This phase uses a more rigorous sampling method as well as an improved scoring function, allowing for more reliable predictions. At the end of this screening process, a total of 36 compounds were selected based on their XP GlideScores, favorable binding poses, and strong interactions with the key residues within the active site. These top hits demonstrated significant binding affinity; with XP GlideScores exceeding −8.0 kcal/mol and the best-scoring compound achieved a GlideScore of −11.2 kcal/mol.

Furthermore, these selected compounds were subjected to a drug-likeness evaluation based on Lipinski’s Rule of Five, using the QikProp module of Schrödinger. Various molecular descriptors were calculated, including molecular weight, logP, hydrogen bond donors and acceptors and number of rotatable bonds. Among the compounds analyzed, eleven of them successfully passed the Lipinski filter without any violations, which suggests favorable oral bioavailability and suitability as potential drug candidates. Detailed information on these compounds and their XP GlideScores are presented in Table 1.

### 2.3. Drug-Likeness and ADME-Tox Profile Assessment

The eleven selected seaweed-derived compounds were subjected to drug-likeness and ADME-Tox evaluation. Their pharmacokinetic and toxicological properties were evaluated using QikProp, SwissADME and Osiris to assess their potential as safe and effective α-glucosidase inhibitors, the results of this analysis were organized in Table 2, Table 3 and Table 4. QikProp provided several key molecular parameters, such as QPlogPo/w (predicted octanol/water partition coefficient), QLogS (predicted aqueous solubility), QLogHERG (predicted HERG channel inhibition risk), QPPCaco (predicted Caco-2 cell permeability), QLogBB (predicted brain/blood partition coefficient), and PSA (polar surface area). These parameters are important indicators of pharmacokinetic properties. All compounds complied with Lipinski’s Rule of Five with no violation (Table 2), which states that drug-like molecules should have a molecular weight ≤ 500 Da, a LogP ≤ 5, no more than 5 hydrogen bond donors, and no more than 10 hydrogen bond acceptors. Likewise, the Rule of Three, advocates for smaller fragments with a molecular weight ≤ 300 Da, a LogP ≤ 3, and no more than 3 hydrogen bond donors and acceptors each. The compliance of all tested compounds with these guidelines support their oral bioavailability. LogP values ranged from −1.17 to 3.77, indicating balanced hydrophilicity/lipophilicity, while solubility (QLogS) remained acceptable despite slightly lower values for some lipophilic molecules. Most compounds showed low risk of cardiotoxicity based on QLogHERG. Caco-2 permeability values indicated good absorption potential for several compounds. Blood–brain barrier permeability values mostly fell between −0.49 and −2.39, indicating limited CNS penetration for most molecules, which may be advantageous for avoiding off-target CNS effects, except for RL495. Additionally, PSA values ranged from 33.49 to 146.16 Å^2^, were within the optimal range for most compounds for good oral bioavailability.

The results in Table 3 suggested that these selected compounds exhibit favorable profiles consistent with drug-like behavior and exhibited a bioavailability score of 0.55, indicating a moderate probability of good oral bioavailability. Regarding gastrointestinal absorption, it is predicted to be high for all compounds, with the exception of BE013. Blood–brain barrier (BBB) permeability is a crucial pharmacokinetic parameter, as compounds intended for the treatment of diabetes do not necessarily require penetration into the central nervous system. Indeed, limited BBB permeability may be advantageous by reducing the risk of potential neurological side effects. This permeability is limited for most compounds except GA009, BD008 and RC002, potentially reducing the risk of central nervous system side effects. In terms of cytochrome P450 enzyme, its inhibition represents an important indicator of the potential risk of drug–drug interactions. Compounds that inhibit specific CYP isoforms may alter the metabolism of co-administered drugs, potentially leading to adverse effects or reduced therapeutic efficacy. GA009 and GA001 are predicted to inhibit multiple CYP isoforms, which could indicate possible drug–drug interactions. Finally, synthetic accessibility scores range from 2.28 (RR021) to 5.39 (BD008), indicating that their synthesis is feasible in a drug development context.

The toxicity assessment (Table 4) revealed that among the eleven evaluated compounds, four molecules RO001, RC002, RR021 and RO006 demonstrated favorable drug scores, indicating an optimal balance between efficacy and safety. These compounds exhibit no predicted toxicity risks, including mutagenic, tumorigenic, irritant and reproductive effects. In particular, RC002 shows the highest drug score (0.85), followed closely by RO001 and RR021 (both 0.75), and RO006 (0.55), making them promising candidates for experimental validation as potential antidiabetic agents derived from marine seaweeds. These characteristics justify their selection as the leading candidates for further investigation in this study.

### 2.4. Protein–Ligand Interactions Analysis

**Figure 2:**
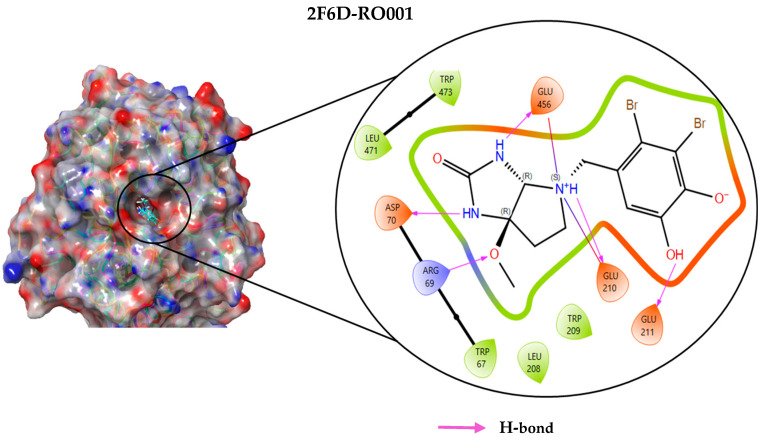
Two-dimensional representation of the interactions between the ligand RO001 and the active site residues of the target protein 2F6D.

**Figure 3:**
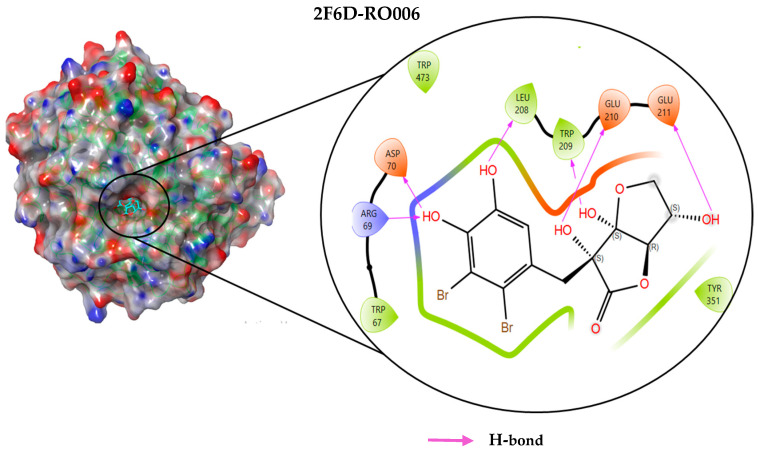
Two-dimensional representation of the interactions between the ligand RO006 and the active site residues of the target protein 2F6D.

**Figure 4:**
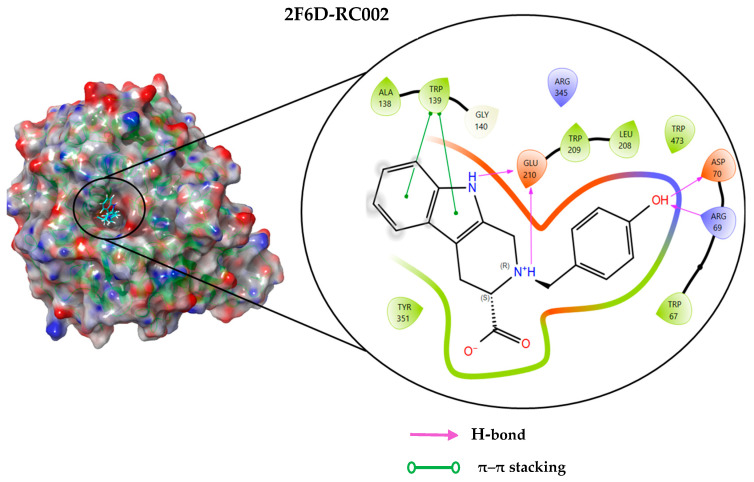
Two-dimensional representation of the interactions between the ligand RC002 and the active site residues of the target protein 2F6D.

**Figure 5:**
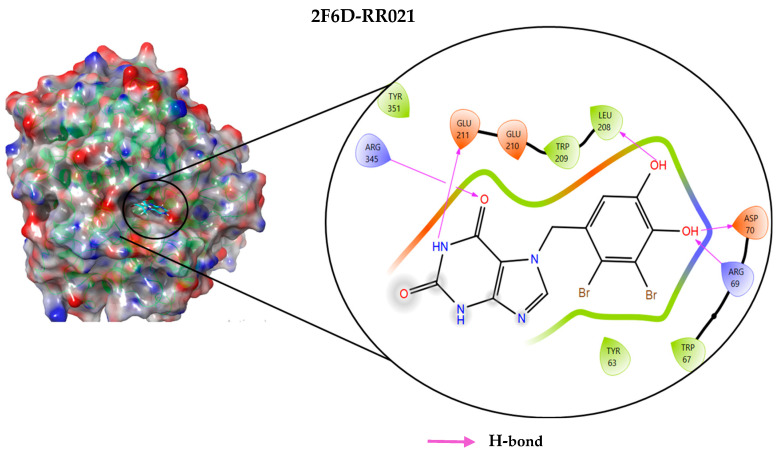
Two-dimensional representation of the interactions between the ligand RR021 and the active site residues of the target protein 2F6D.

The analysis of interactions between the ligands and the active site residues is essential to understand the enzyme inhibition mechanism, as the nature and stability of these interactions largely determine the inhibitory effectiveness of the compounds. From the eleven compounds, four top promising molecules: RO001 (Colensolide A), and RO006 (Rhodomelol), both isolated from the red alga *Osmundaria colensoi* collected in Northland, New Zealand. RC002 (Callophycin A), derived from the red alga *Callophycus oppositifolius* found in Pugh Shoal, Truant Island, Australia. And RR021 (7-(2,3-dibromo-4,5-dihydroxybenzyl)-3,7-dihydro-1*H*-purine-2,6-dione), obtained from the red alga *Rhodomela confervoides* sampled in the Gulf of the Yellow Sea, China, were selected for an in-depth analysis of their interactions with the 2F6D protein. The results of this analysis are presented in Table 5 and Figure 2, Figure 3, Figure 4 and Figure 5.

Compound RO001 (Figure 2) formed five hydrogen bonds with residues Arg69, Asp70, Glu210, Glu211 and Glu456, with a binding score of −10.58 kcal/mol. RO006 (Figure 3) also showed a favorable docking score of −8.817 kcal/mol, forming six hydrogen bonds with Arg69, Asp70, Leu208, Trp209, Glu210 and Glu211. RC002 (Figure 4) achieved a docking score of −8.493 kcal/mol. It formed four hydrogen bonds with Arg69, Asp70 and Glu210, and two π–π stacking interactions with Trp139. The presence of aromatic stacking interactions enhances the ligand’s stabilization in the hydrophobic region of the binding pocket. And for RR021 (Figure 5), it recorded an XP score of −8.113 kcal/mol and engaged in five hydrogen bonds with residues Arg69, Asp70, Leu208, Glu211 and Arg345. As previously shown (Figure 2), acarbose formed multiple hydrogen bonds with Arg69, Asp70, Gly140, Leu208, Trp209, Glu210, and Glu211. This indicates that the interactions formed by the selected molecules resemble those formed by acarbose, suggesting that they may play a similar role in inhibiting α-glucosidase activity. All of these interactions were established with the active site residues of the protein, demonstrating their inhibitory effect. By reducing carbohydrate degradation, these compounds exhibit a promising mechanism of action aimed at decreasing glucose absorption in the intestine, which could effectively contribute to glycemic control in patients with type 2 diabetes.

### 2.5. DFT Study

**Figure 6:**
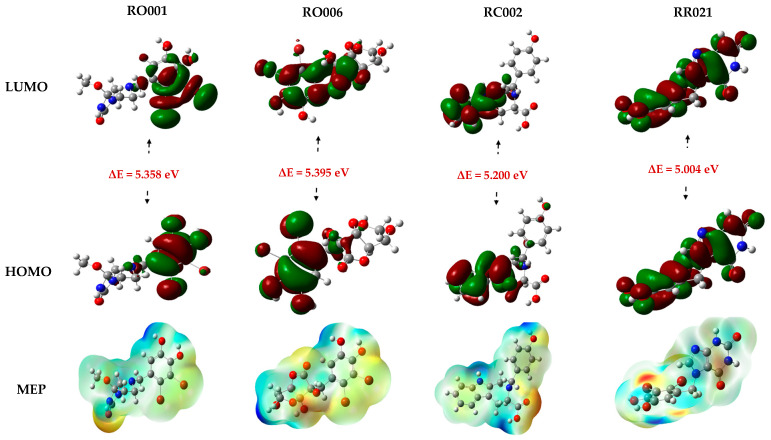
Distribution of the frontier molecular orbitals HOMO, LUMO and molecular electrostatic potential maps for the four selected compounds.

To gain further insight into the electronic properties and reactivity of the selected ligands, Density Functional Theory calculations were performed. The results are presented in Table 6 and Figure 6. The spatial distribution of the frontier molecular orbitals HOMO and LUMO provides valuable insights into the regions of the molecule involved in electronic interactions, highlighting the main electron-donating and electron-accepting sites. For RO001, both the HOMO and LUMO are delocalized over the aromatic ring containing hydroxyl groups and bromine atoms. In RO006, these orbitals are distributed over nearly the entire molecule, with the exception of the aromatic ring. For RC002, they are primarily localized on the indole ring, highlighting this moiety as the key contributor to its electronic activity and interactions. Lastly, in RR021, both orbitals cover almost the entire molecule, reflecting a high degree of electron delocalization. Analysis of quantum parameters showed that the gap energy values varies from 5.004 eV (RR021) to 5.395 eV (RO006), indicating enhanced electronic stability. In terms of electronegativity (χ), RR021 has the highest value, indicating a stronger tendency to attract electrons. The chemical potential (µ) is more negative for RR021 (−3.588 eV), which may favor spontaneous electron donation in biological systems. The electrophilicity index (ω) reflects the ability of the molecule to accept electrons, RO001 and RR021 show relatively high values (ω = 2.125 and 2.572 eV, respectively), suggesting good electron-accepting capability. The relatively low softness values, ranging from 0.371 to 0.400 eV^−1^, suggest that these molecules may exhibit a certain degree of flexibility, allowing them to adapt their conformation within the active site of the target protein, which could enhance binding interactions. Finally, the dipole moment gives insight into molecular polarity. RO001 displays the highest dipole moment (8.463 D), which implies stronger polarity and may enhance solubility and binding interactions in biological environments. Conversely, RC002 has the lowest dipole moment (5.081 D).

The MEP maps of these selected compounds highlight the distribution of electron density, allowing the identification of regions prone to electrophilic and nucleophilic interactions. Red regions, which correspond to zones of high electron density, are predominantly located around electronegative atoms such as oxygen and bromine, suggesting favorable sites for electrophilic attack. In contrast, blue regions, indicative of electron-deficient areas, are mainly observed around hydrogen atoms, particularly those bonded to electronegative atoms like oxygen and nitrogen, which could serve as potential sites for nucleophilic interactions. This distribution of electrostatic potential highlights the possible interaction modes of the molecules within the active site of the target protein and may influence their binding affinity.

### 2.6. Molecular Dynamics Analysis

**Figure 7:**
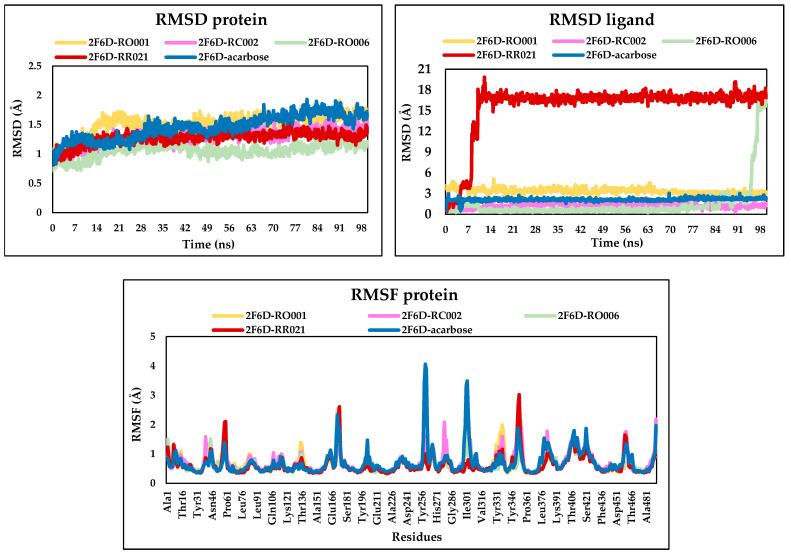
RMSD and RMSF plots of the simulated complexes after 100 ns of molecular dynamics.

**Figure 8:**
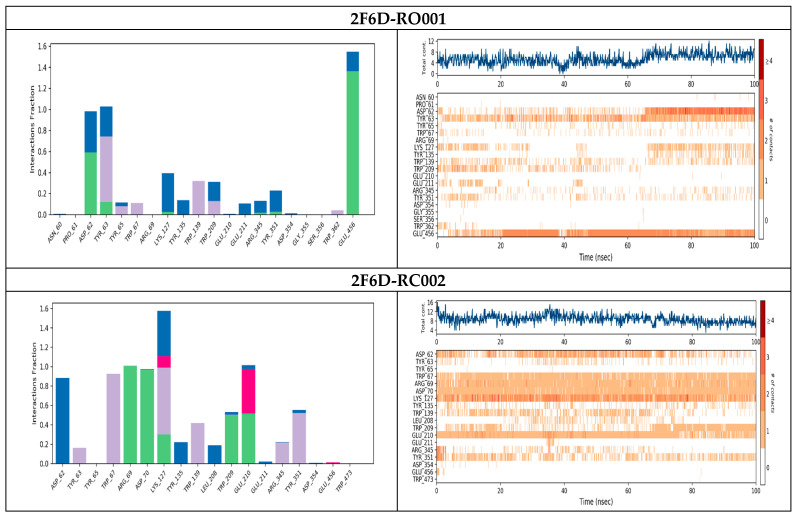

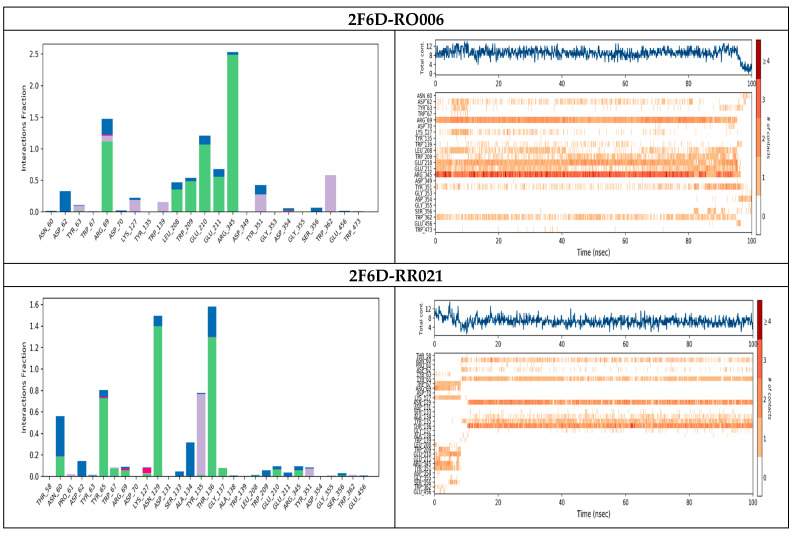
Protein–ligand contact, histogram and interaction timeline of the simulated complexes. Colors indicate interaction types: green for hydrogen bonds, pink for ionic bonds, violet for hydrophobic interactions, and blue for water bridges.

Molecular dynamics simulations were performed for the top-selected compounds and the reference drug in complex with the 2F6D receptor. Each simulation was run for 100 ns, generating trajectory data for every complex. Key parameters such as root mean square deviation and root mean square fluctuation were calculated and presented in Figure 7. Additionally, the interactions formed between the ligands and the receptor were analyzed to evaluate their persistence over time, providing insights into both the stability and strength of the ligand–receptor complexes (Figure 8).

The calculated protein RMSD values for each receptor-ligand complex, showing overall good stability throughout the 100 ns simulation. The RMSD values for all complexes fluctuated within a narrow range of approximately 0.8 to 1.8 Å, specifically, the selected ligands showed lower values compared to acarbose, indicating a good stability of the protein structure in the presence of these ligands and minimal structural deviation over time.

For the ligand RMSD, the ligands RO001 and RC002 exhibited a remarkably stable behavior throughout the entire simulation period compared to the reference drug, with average RMSD values of approximately ranging between 1 Å and 3.7 Å. For RO006, it exhibited a greater stability within the binding pocket, as evidenced by low RMSD values (0.8–1 Å) maintained up to 95 ns. A subsequent increase reaching approximately 15 Å indicates a movement of the ligand within the active site toward the end of the simulation. In contrast, RR021 displayed instability from the early stages of the simulation, reflected by an increase in RMSD values, suggesting that the ligand was not stably retained in the binding site and may have undergone partial displacement or reorientation in the protein.

RMSF analysis provides insight into the individual flexibility of protein residues during the simulations. The RMSF plots reveal minor fluctuations across most residues for the all complexes, with RMSF values remaining below 3 Å. Two notable peaks were observed at Asp261 and Asp300 for the complex 2F6D-acarbose, indicating localized flexibility. Overall, the low fluctuation levels suggest that the amino acid residues attained stability upon interaction with the complexed ligands.

The molecular interactions between the ligands and the 2F6D receptor are illustrated in Figure 8, which includes both the protein–ligand contact, the interaction histogram and timline. The 2F6D-RO001 complex exhibited three key interactions: two hydrogen bonds with Asp62 and Glu456, and a hydrophobic interaction with Tyr63, which together contribute to the observed stability and specificity of the ligand binding within the active site throughout the simulation and also contributing significantly to the overall structural integrity and potential inhibitory effectiveness of the ligand. For the 2F6D–RC002 complex, the high stability previously observed in the RMSD analysis can be attributed to the formation of multiple interactions, particularly strong hydrogen bonds with residues Arg69, Asp70, and Glu210, as well as hydrophobic contacts with Thr67 and Lys127. According to the contact timeline, these interactions remained stable, strong, and persistent throughout the simulation, which supports the robustness of the complex. In the case of the 2F6D–RO006 complex, hydrogen bonding predominated, especially with residues Arg69, Leu208, Trp209, Glu210, Glu211, and Arg345. These interactions contributed to a stable binding mode maintained up to approximately 95 ns, further confirming the compound’s affinity and stability within the active site. Conversely, for the 2F6D–RR021 complex, a loss of the initial docking interactions was observed early in the simulation. New contacts were established with Tyr65, Asn129, Tyr135, and Thr136, suggesting that the ligand shifted from its original binding site. This displacement correlates with the early increase in RMSD values, indicating reduced stability and weaker anchoring within the active pocket.

### 2.7. Limitations of the Study

Although the present study provides valuable insights into the potential inhibitory activity of marine algae-derived natural compounds against α-glucosidase using an integrated in silico approach, certain limitations inherent to the applied methodology should be acknowledged for a rigorous interpretation of the results. First, molecular docking, while effective for predicting binding modes, relies on a largely rigid protein model and does not fully account for protein flexibility, which may influence ligand–enzyme interactions under physiological conditions. Second, although molecular dynamics simulations were conducted to evaluate the stability of the selected protein–ligand complexes, extending these simulations over longer time scales and incorporating additional analyses could provide deeper insights into long-term conformational behavior. Finally, as this investigation is purely computational, experimental validation remains essential. The predicted inhibitory potential of the identified compounds must be confirmed through in vitro assays and in vivo studies to establish their actual biological efficacy.

## 3. Materials and Methods

### 3.1. Data Source

A natural compounds library derived from marine seaweeds, containing 1191 unique ligands, was downloaded from the Seaweed Metabolite Database https://www.swmd.co.in/ (accessed on 22 May 2025) [36]. This comprehensive dataset includes metabolites belonging to 884 taxa of red algae (Rhodophyta), 274 taxa of brown algae (Phaeophyta) and 33 taxa of green algae (Chlorophyta). All seaweed compounds were retrieved in three-dimensional Protein Data Bank (PDB) format, allowing their use in structure-based virtual screening.

### 3.2. Preparation of Ligands

The pre-optimized ligands were prepared using the LigPrep tool, employing the OPLS3e (Optimized Potentials for Liquid Simulations) force field [37]. The library was energy-minimized. The software was set up to generate up to 5 conformers for each ligand, with states generated at a pH of 7 ± 2 [38].

### 3.3. Selection and Preparation of Protein

**Figure 9:**
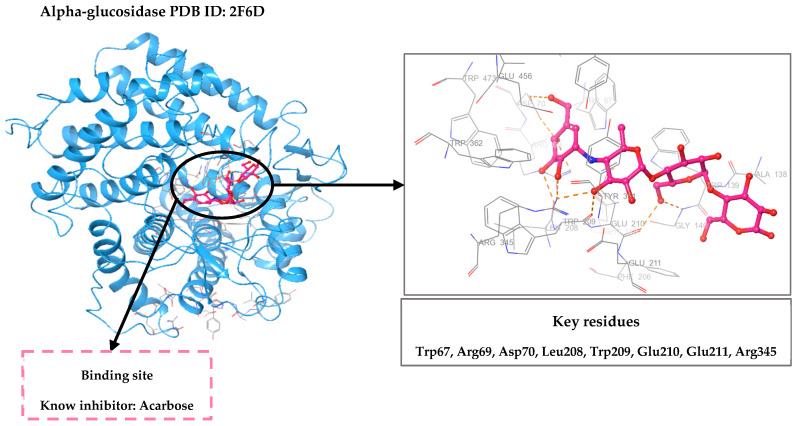
Three-dimensional structure of alpha-glucosidase (PDB ID: 2F6D), co-crystallized with Acarbose. The active site is occupied by the reference inhibitor Acarbose, highlighting key interaction regions.

The crystal structure of alpha-glucosidase (PDB code: 2F6D) [39] (Figure 9) was downloaded from the Protein Data Bank (www.rcsb.org accessed on 25 May 2025). The choice of this specific structure was influenced by its high resolution of 1.60 Å, as well as its complexation with Acarbose, a recognized inhibitor of alpha-glucosidase [40]. This structure consists of 492 amino acids. It also includes heteroatoms such as Acarbose, phosphate groups and sodium ions. As shown in Figure 9, the 3D structure of this protein reveals that the active site is occupied by Acarbose, thereby delineating the key interaction regions within the binding pocket.

Before proceeding with molecular docking, the protein structure was prepared using the Protein Preparation Wizard tool. This step involved correcting structural anomalies, including atom overlaps, alternative positions, missing atoms, and incorrect atom types, all of which were identified and rectified by minimizing the structure and repairing missing side chains. Water molecules were removed, and hydrogen atoms were added at a pH = 7.4 [38].

### 3.4. Molecular Docking

The receptor grid for the protein was generated using the Receptor Grid Generation tool in Maestro, based on the position of the co-crystallized ligand, Acarbose, in the protein structure, with the following coordinates x = 12.68 Å, y = 10.80 Å, z = −6.35 Å. Default parameters were used for adjusting the van der Waals radius (scaling factor = 1, partial charge cutoff = 0.25). The studied compounds were docked into the active site of the protein using three Glide docking approaches: HTVS, SP and XP. These modes differ in their precision, speed, and evaluation functions. The HTVS mode allows for rapid screening, reducing intermediate conformations. The SP mode strikes a balance between speed and accuracy, typically requiring about 10 s per compound. Moreover, the XP mode aims to reduce false positives by penalizing molecules with low binding affinity to the receptor [41].

### 3.5. Drug-likeness and ADMET Filtering

The drug-likeness and pharmacokinetic properties of the selected compounds were evaluated using two computational tools: SwissADME web server [42] and QikProp from the Schrödinger suite [43,44]. These tools provide comprehensive predictions related to Lipinski’s Rule of Five, gastrointestinal (GI) absorption, blood–brain barrier (BBB) permeability, and various other pharmacokinetic parameters essential for early drug development. In addition, the Osiris Property Explorer was employed to assess the toxicity risk of the molecules, including potential mutagenicity, tumorigenicity, irritant effects, and reproductive toxicity.

### 3.6. Molecular Dynamics Simulations

The molecular dynamics (MD) was performed to evaluate the stability of complexes formed between the protein 2F6D and the potential compounds, using Maestro-Desmond v2020-3 software [44]. After initial preparation and structural optimization, the complexes underwent energy minimization using the OPLS3e force field [37]. The systems were solvated using the TIP3P water model [45] in a cubic simulation box (10 Å × 10 Å × 10 Å) and neutralized by adding Na^+^ and Cl^−^ ions to a concentration of 0.15 M. The simulations started with a 1 ns NVT equilibration phase, followed by 100 ns under an NPT ensemble at a constant temperature of 300 K and pressure of 1 atm. The stability of the systems was assessed using several parameters, including root-mean-square deviation (RMSD), root-mean-square fluctuation (RMSF) [46], and protein–ligand contacts.

### 3.7. DFT Calculations

Density Functional Theory was used to evaluate the electronic properties of the best molecules based on the distribution of their electron density [47]. The structures of the studied compounds were first modeled in three dimensions using the visualization software GaussView 6.0 [48] and then optimized using the Gaussian 09 program [49]. The optimization process utilized the three-parameter hybrid models of Becke and the Lee, Yang and Parr (B3LYP) correlation functional [50,51], using a 6-31G(d,p) basis set [52]. The calculations were performed in water as solvent using the CPCM solvent model, while the vibrational analysis was performed to verify the presence of energy minima, indicated by the absence of imaginary frequencies. The frontier molecular orbitals, namely the Highest Occupied Molecular Orbital and the Lowest Unoccupied Molecular Orbital, were calculated using the same level of theory. These calculations were also employed to determine various quantum parameters using the following formulas:
**Ionization potential****Electron affinity****Energy gap**IP = −E_HOMO_EA = −E_LUMO_ΔE= E_LUMO_ − E_HOMO_**Hardness****Softness****Electronegativity**η = (IP − EA)/2σ = 1/ηχ = (IP + EA)/2**Chemical potential****Electrophilicity index**μ = − χω = μ^2^/2η

Molecular Electrostatic Potential (MEP) analysis was carried out to identify regions in the selected compounds that are likely to undergo electrophilic and nucleophilic attacks [53].

## 4. Conclusions

This study investigated the antidiabetic potential of a library of natural compounds derived from marine seaweeds, targeting the α-glucosidase enzyme through a structure-based virtual screening strategy. An integrative in silico approach was employed, combining molecular docking, ADMET prediction, molecular dynamics simulations and Density Functional Theory calculations. Based on XP docking scores and drug-likeness filters, eleven molecules were initially selected. Following further evaluation of their pharmacokinetic and toxicity profiles, four promising lead compounds were identified from the Seaweed Metabolite Database: RO001 (Colensolide A), and RO006 (Rhodomelol), both isolated from the red alga *Osmundaria colensoi*. RC002 (Callophycin A), derived from the red alga *Callophycus oppositifolius* And RR021 (7-(2,3-dibromo-4,5-dihydroxybenzyl)-3,7-dihydro-1*H*-purine-2,6-dione), obtained from the red alga *Rhodomela confervoides*. These compounds demonstrated effective inhibitory potential against the α-glucosidase enzyme with a binding affinity ranging between −8.113 and −10.58 kcal/mol and they formed a strong interaction with the key residues of the active site. ADMET predictions confirmed their favorable pharmacokinetic and toxicity profiles. DFT analysis provided insights into their electronic properties and molecular reactivity, MD simulations confirmed the stability of the complexes formed with RO001, RO006 and RC002, whereas the RR021 ligand did not exhibit stable binding throughout the simulation. Overall, these findings underline the therapeutic promise of seaweed-derived compounds and support their further development as novel antidiabetic agents. Future work will focus on evaluating their biological activity through in vitro and in vivo studies to validate their efficacy.

## Figures and Tables

**Table 1:** Chemical structures, detailed information and XP docking scores of the top selected ligands.

Molecule	Name	Source	Structure	XP Score (kcal/mol)	Molecule	Name	Source	Structure	XP Score (kcal/mol)
**RO001**	Colensolide A	***Osmundaria colensoi***	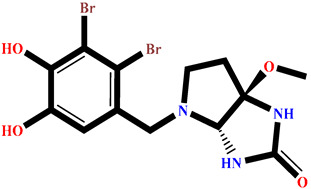	**−10.58**	**BD008**	8α,11-dihydroxypachydictyol A	***Dictyota* sp. **	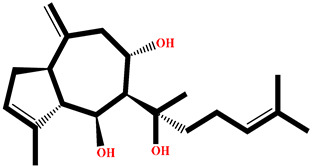	**−8.459**
**BE013**	Eckstolon	***Ecklonia*** ***stolonifera***	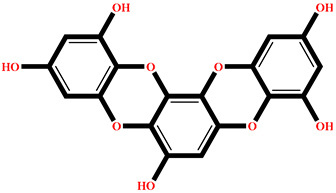	**−9.708**	**GA001**	5-hydroxyisoavrainvilleol	***Avrainvillea nigricans***	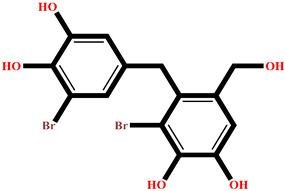	**−8.367**
**RR023**	(2S)-2-amino-3-(3-bromo-5-hydroxy-4-methoxyphenyl)propanoic acid	***Rhodomela confervoides***	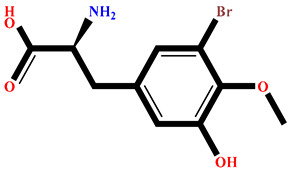	**−9.304**	**RL495**	2-methylsulfinyl-3-methylthio-4,5,6-tribromoindole	***Laurencia brongniartii***	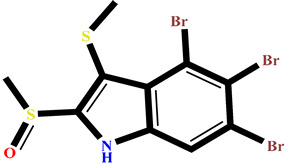	**−8.233**
**RO006**	Rhodomelol	***Osmundaria colensoi***	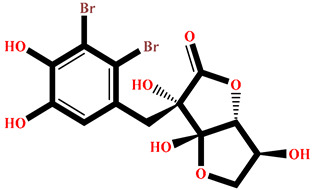	**−8.817**	**RR016**	*N*-(2,3-Dibromo-4,5-dihydroxybenzyl)methyl pyroglutamate	***Rhodomela confervoides***	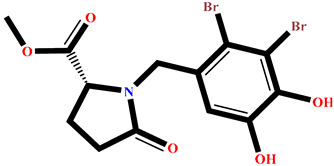	**−8.163**
**RC002**	Callophycin A	***Callophycus*** ***oppositifolius***	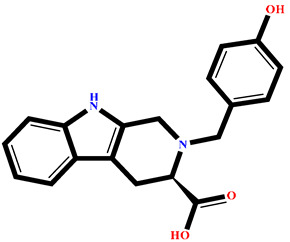	**−8.493**	**RR021**	7-(2,3-dibromo-4,5-dihydroxybenzyl)-3,7-dihydro-1*H*-purine-2,6-dione	***Rhodomela*** ***confervoides***	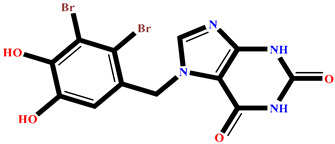	**−8.113**
**GA009**	Avrainvilleol methyl ether	***Avrainvillea rawsonii***	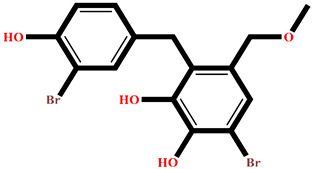	**−8.462**	**Reference** **drug**	Acarbose	**---**	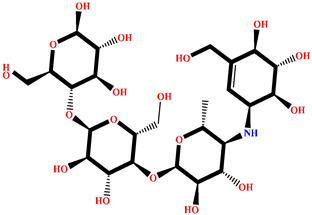	**−12.33**

**Table 2:** Predicted ADME properties of the eleven selected compounds calculated using the QikProp module.

Molecule	QPlogPo/w	QLogS	QLogHERG	QPPCaco	QLogBB	QLogKp	PSA	Rule of Five	Rule of Three
RO001	0.952	−2.505	−4.93	40.40	−0.79	−6.38	110.72	0	0
BE013	0.825	−3.546	−5.847	26.89	−2.39	−4.95	141.37	0	0
RR023	−1.168	−1.438	−2.40	11.44	−0.75	−6.17	101.45	0	1
RO006	0.030	−2.585	−3.73	48.51	−1.68	−5.17	146.16	0	0
RC002	0.730	−3.811	−4.66	18.06	−0.82	−5.03	86.91	0	1
GA009	3.164	−4.212	−4.74	692.88	−0.63	−2.57	69.93	0	0
BD008	3.779	−4.363	−4.22	2154.73	−0.49	−1.84	49.94	0	1
GA001	1.373	−2.974	−4.29	72.11	−1.64	−4.52	105.52	0	0
RL495	3.357	−3.343	−4.11	104.45	0.44	−2.19	33.49	0	0
RR016	0.903	−1.913	−1.94	144.31	−0.88	−3.98	100.97	0	0
RR021	0.425	−3.105	−3.88	27.91	−1.71	−5.77	143.99	0	0

**logPo/w**: octanol/water partition coefficient; **LogS**: solubility; **LogHERG**: inhibition of hERG; **PCaco**: Caco-2 cell permeability; **LogBB**: brain/blood partition coefficient; **LogKp**: skin permeability; **PSA**: Polar surface area.

**Table 3:** Physicochemical properties, predicted pharmacokinetics, and drug-likeness of the eleven selected compounds based on SwissADME analysis.

Molecule	RO001	BE013	RR023	RO006	RC002	GA009	BD008	GA001	RL495	RR016	RR021
Formula	C_13_H_15_Br_2_N_3_O_4_	C_18_H_10_O_9_	C_10_H_12_BrNO_4_	C_13_H_12_Br_2_O_8_	C_19_H_18_N_2_O_3_	C_15_H_14_Br_2_O_4_	C_20_H_32_O_3_	C_14_H_12_Br_2_O_5_	C_10_H_8_Br_3_NOS_2_	C_13_H_13_Br_2_NO_5_	C_12_H_8_Br_2_N_4_O_4_
MW	437.08	370.27	290.11	456.04	322.36	418.08	320.47	420.05	462.02	423.05	432.02
Rotatable bonds	3	0	4	2	3	4	4	3	2	4	2
H-bond acceptors	5	9	5	8	4	4	3	5	1	5	5
H-bond donors	4	5	3	5	3	3	3	5	1	2	4
Log P	1.26	2.05	−1.25	0.02	1.79	3.01	2.85	2.21	3.76	1.53	1.05
GI absorption	High	Low	High	High	High	High	High	High	High	High	High
BBB permeant	No	No	No	No	Yes	Yes	Yes	No	No	No	No
CYP1A2 inhibitor	No	No	No	No	No	Yes	No	Yes	No	Yes	No
CYP2C19 inhibitor	Yes	No	No	No	No	Yes	No	No	No	Yes	No
CYP2C9 inhibitor	No	No	No	No	No	Yes	No	Yes	Yes	No	No
CYP2D6 inhibitor	No	No	No	No	Yes	Yes	No	Yes	No	No	No
CYP3A4 inhibitor	No	No	No	No	No	Yes	No	Yes	Yes	No	No
Lipinski violations	0	0	0	0	0	0	0	0	0	0	0
Bioavailability Score	0.55	0.55	0.55	0.55	0.55	0.55	0.55	0.55	0.55	0.55	0.55
Synthetic Accessibility	3.46	3.48	2.41	4.09	2.96	2.73	5.39	2.59	3.09	2.68	2.28

**MW**: Molecular weight; **GI absorption**: gastrointestinal absorption; **BBB**: blood–brain barrier; **CYP inhibitors**: Potential inhibition of cytochrome P450 isoforms.

**Table 4:** Toxicity risk assessment for the eleven selected compounds predicted using OSIRIS Property.

Molecule	Toxicity Risk				Drug Likeness	Drug Score
	Mutagenic	Tumorigenic	Irritant	Reproductive Effective		
RO001	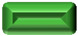	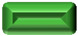	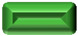	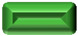	2.35	0.75
BE013	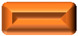	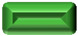	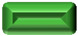	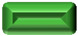	−1.28	0.21
RR023	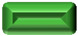	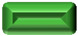	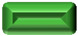	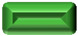	−10.96	0.47
RO006	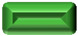	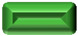	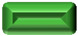	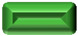	−0.43	0.55
RC002	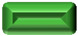	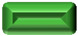	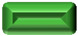	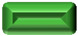	2.44	0.85
GA009	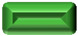	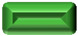	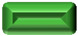	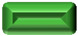	−2.59	0.35
BD008	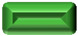	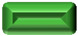	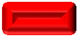	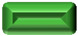	−2.6	0.24
GA001	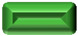	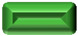	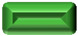	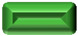	−2.74	0.39
RL495	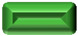	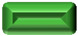	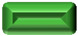	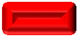	−1.13	0.2
RR016	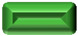	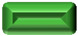	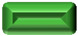	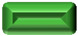	−1.27	0.49
RR021	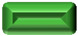	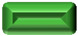	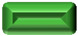	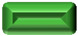	2.98	0.75
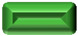	No risk	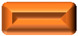	Moderate risk	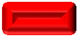	High risk	

**Table 5:** Key interactions formed between the four selected compounds and acarbose, and the active site residues of 2F6D protein, involved residues and interaction type.

Complex	XP Score (kcal/mol)	Involved Residues	Interaction Type
2F6D-RO001	−10.580	Arg69, Asp70, Glu210, Glu211, Glu456	Hydrogen bond
2F6D-RO006	−8.817	Arg69, Asp70, Leu208, Trp209, Glu210, Glu211	Hydrogen bond
2F6D-RC002	−8.493	Arg69, Asp70, Glu210 Trp139	Hydrogen bond π–π stacking
2F6D-RR021	−8.113	Arg69, Asp70, Leu208, Glu211, Arg345	Hydrogen bond
2F6D-Acarbose	−12.330	Arg69, Asp70, Gly140, Leu208, Trp209, Glu210, Glu211	Hydrogen bond

**Table 6:** Quantum descriptors derived from DFT calculations for the four selected compounds, using the B3LYP/6-31G(d,p) level.

Molecules	HOMO (eV)	LUMO (eV)	ΔE (eV)	χ (eV)	η (eV)	µ (eV)	σ (eV^−1^)	ω (eV)	Dipol Moment (D)
RO001	−6.054	−0.696	5.358	3.375	2.679	−3.375	0.373	2.125	8.463
RO006	−5.871	−0.476	5.395	3.174	2.698	−3.174	0.371	1.867	7.864
RC002	−5.529	−0.329	5.200	2.929	2.600	−2.929	0.385	1.650	5.081
RR021	−6.090	−1.086	5.004	3.588	2.502	−3.588	0.400	2.572	7.560

## Data Availability

No new data were created or analyzed in this study.
